# Flexural Capability of Patterned Transparent Conductive Substrate by Performing Electrical Measurements and Stress Simulations

**DOI:** 10.3390/ma9100850

**Published:** 2016-10-19

**Authors:** Chang-Chun Lee, Pei-Chen Huang, Ko-Shun Wang

**Affiliations:** 1 Department of Mechanical Engineering, National Chung Hsing University, Taichung City 40227, Taiwan; mars420225@gmail.com; 2 iboson Technology Corporation, Chubei City 30268, Taiwan; kersonwang@i-boson.com

**Keywords:** patterned ITO/PET film, bending test, sheet resistance, bending strain, finite element analysis

## Abstract

The suitability of stacked thin films for next-generation display technology was analyzed based on their properties and geometrical designs to evaluate the mechanical reliability of transparent conducting thin films utilized in flexural displays. In general, the high bending stress induced by various operation conditions is a major concern regarding the mechanical reliability of indium–tin–oxide (ITO) films deposited on polyethylene terephthalate (PET) substrates; mechanical reliability is commonly used to estimate the flexibility of displays. However, the pattern effect is rarely investigated to estimate the mechanical reliability of ITO/PET films. Thus, this study examined the flexible content of patterned ITO/PET films with two different line widths by conducting bending tests and sheet resistance measurements. Moreover, a stress–strain simulation enabled by finite element analysis was performed on the patterned ITO/PET to explore the stress impact of stacked film structures under various levels of flexural load. Results show that the design of the ITO/PET film can be applied in developing mechanically reliable flexible electronics.

## 1. Introduction

Flexible electronic devices based on polymer substrates, such as organic light-emitting diodes (OLED), cholesteric liquid crystal displays (ChLCDs), and thin film transistors (TFT) are generally regarded as promising candidates for next-generation electronic devices. The brittle indium–tin–oxide (ITO) film is the transparent conducting material that is most widely used as anode in OLED devices. The advantages of this material include low electrical resistance, high light transmission characteristics, and easy manufacture in large areas by roll-to-roll sputter-coating and printing techniques [1,2,3]. Transparent conducting films, such as ITO and zinc oxide (ZnO), easily undergo brittle failure or cracking when subjected to bending load. The nano-indentation technique was introduced to investigate the mechanical properties of these conducting films [4,5]. To optimize the structural design of multi-stacked thin films, Chen et al. proposed an analytical model for the strain-isolation design of flexible devices; their results indicated that the insertion of a soft film with ultra-low Young’s modulus as the isolation layer can protect the device against substrate deformation under a bending load [6]. Wang et al. performed a simulation by finite element analysis (FEA) to obtain the residual, intrinsic, and thermal stresses in a multilayered structure after annealing [7]. The brittle fracture of thin transparent conducting films is a drawback for the mechanical reliability of flexible devices. Zhen et al. proposed novel experimental methods to investigate the fracture properties and mechanisms of ITO/polyethylene terephthalate (PET) films under tensile/compressive stress; their results showed that cracking occurred under tensile stress, and that buckling delamination was observed when compressive stress was introduced [8,9]. The fatigue life of ITO films deposited on PET compliant substrate, on which various pre-strain levels were applied, was investigated by ANOVA. The effects of bending frequency, bending diameter, number of bending cycles, and sample width on resistance change and residual stress were systemically investigated [10,11,12]. Moreover, the mechanical stability of ITO/PET films with different interlayer designs, strain rates, and substrate materials was also examined [13,14,15,16,17,18]. Using a low mechanical stiffness can prevent the failure of ITO films, and finding the suitable combination of multi-thin films can reduce the influence of external loading on ITO films [19]. Moreover, Wen et al. proposed two key mechanical loading types—namely, fatigue bending and torsion—to investigate the failure mechanism and optical characteristics of flexible ChLCD panels under different loading conditions [20]. However, limited attention has been devoted to the pattern effect on the mechanical reliability of ITO/PET films, which affects the stress–strain distribution of the stacked thin film structure. Thus, this study investigated patterned ITO/PET films with two types of pattern design. Resistance change under different levels of bending radius of curvature was obtained to estimate the electrical properties under a bending load. Moreover, the stress–strain distribution was simulated using a FEA approach to estimate the failure possibility and critical location of the patterned ITO/PET film.

## 2. Experimental Details of Patterned ITO/PET Films

### 2.1. Establishment of the Flexible Characteristic Inspection System and the Bending Experiment

**Figure 1:**
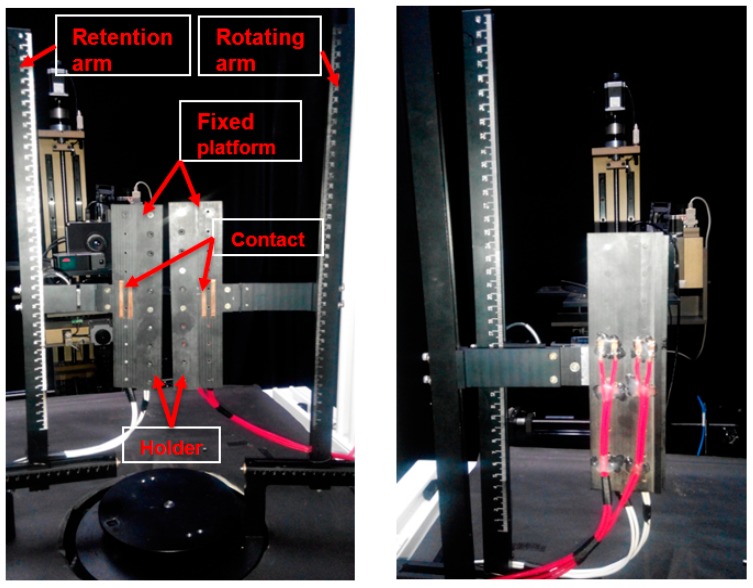
Flexible characteristic inspection system utilized in the patterned indium–tin–oxide/polyethylene terephthalate (ITO/PET) bending experiment.

**Figure 2:**
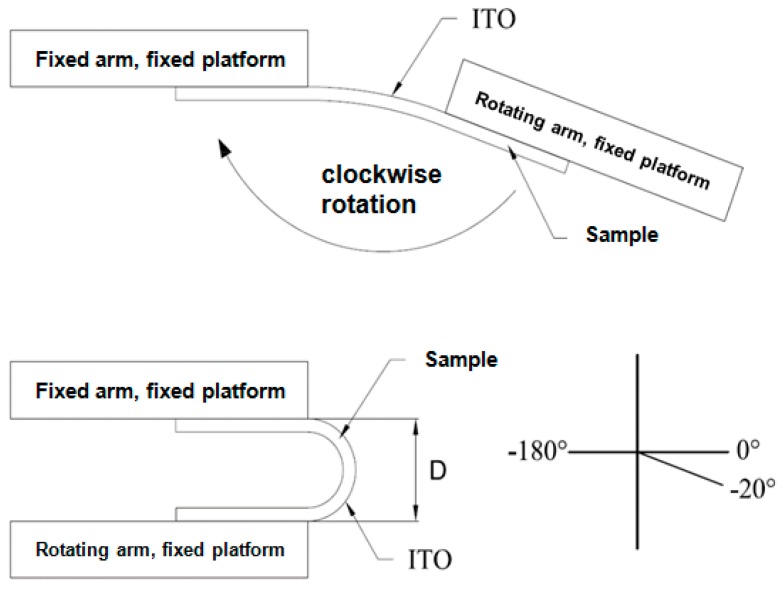
Schematic diagram of operating bending tester.

The bending testing mechanism utilized in this study is shown in Figure 1. The system consists of two fixed platforms and a holder to fix the sample during the experiment. Moreover, a four-point probe was installed on the platform to measure the electrical resistance of the ITO/PET film under bending test. After the testing sample was placed on the platform, the retention arm was fixed, and only the rotating arm was operated during the bending test. The operating mechanism of the bending tester is shown in Figure 2. The testing sample was rotated 20° on the fixed platform to ensure good contact between the sample and the platform. In this way, the bending test of patterned ITO/PET film can be performed.

### 2.2. Specimen Structure and Manufacture Process of Patterned ITO/PET Film

**Figure 3:**
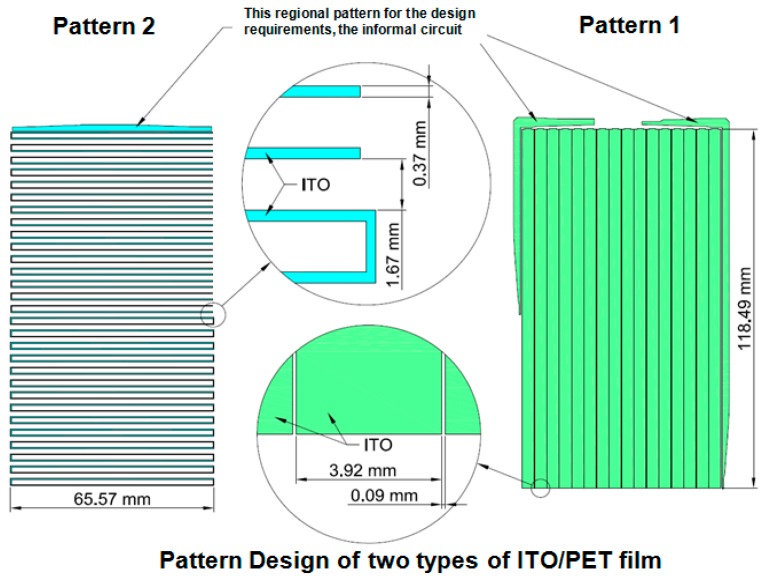
Top view of two types ITO/PET film specimens.

**Figure 4:**
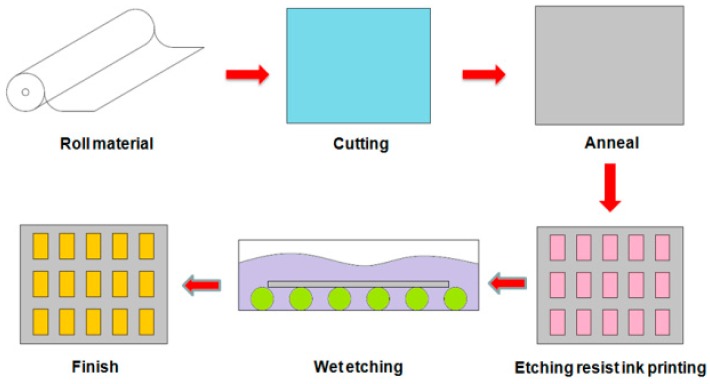
Manufacture processes of patterned ITO/PET film.

As shown in Figure 3, the testing sample had the following geometry: 118.49 mm × 65.57 mm (length × width) area, pattern 1 sample with 3.92 mm line width and 0.09 mm gap, pattern 2 sample with 0.37 mm line width and 1.67 mm gap. To distinguish the two types of patterned ITO/PET films and for convenience of discussion, the patterned ITO/PET films with 3.92 and 0.37 mm line width are named pattern 1 and pattern 2 ITO/PET film, respectively. Figure 4 illustrates the manufacture process of the patterned ITO/PET films utilized in the experimental study. The ITO films were subjected to rolling, cutting, and annealing to ensure the excellent electrical properties, and to release the residual stress of the film. The testing specimen patterns were prepared by depositing two types of patterned ITO films on PET substrates with three different thicknesses of 50, 100, and 125 μm. Finally, wet etching was performed to finish the manufacture process of the patterned ITO/PET film.

## 3. Experimental Results and Discussion

**Figure 5:**
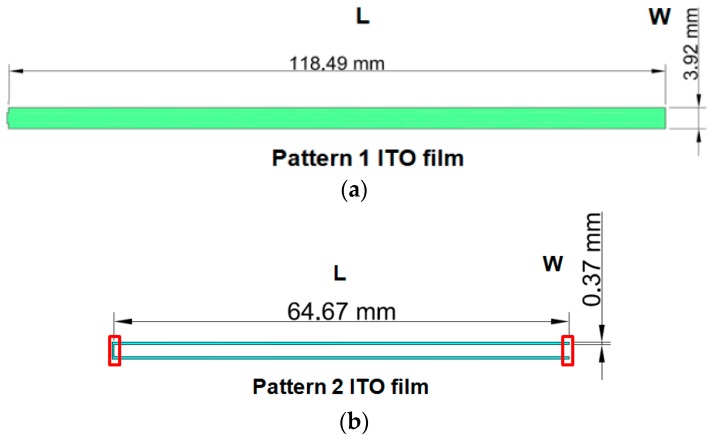
Testing sample of patterned ITO/PET film: (**a**) pattern 1 ITO film (ITO width = 3.92 mm); (**b**) pattern 2 ITO film (ITO width = 0.37 mm).

To explore the pattern effect of ITO coating, test samples with two different patterns were cut along the length direction of the ITO lines, as shown in Figure 5. A four-point probe was installed to measure the sheet resistance and patterned sample resistance of the ITO/PET films when the sample was at a flat position to ensure that the cutting process would not induce any damage. Moreover, the theoretical value was calculated using the following equation:

(1) $$R=R_{s}\frac{L}{W}$$

where *R* and *R_s_* refer to the calculated resistance and sheet resistance, respectively. Symbols *L* and *W* are separately denote the pattern length and width.

Table 1 lists the measured and calculated electrical characteristics of all tested samples with two different patterns and three various PET thicknesses (i.e., 50, 100, and 125 µm). The measured electrical resistance of the pattern 2 ITO/PET film was significantly larger than that of the pattern 1 ITO film because of the narrow line width of the pattern 2 sample. The experimental data of the patterned ITO/PET films agree well with the analytical solution adopted in this study. Thus, reliable sample preparation and testing methods were introduced.

**Figure 6:**
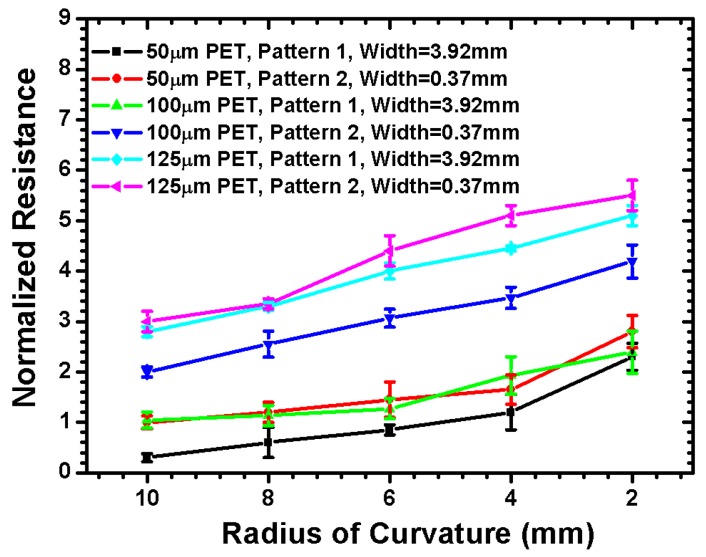
Normalized electrical resistance of patterned ITO/PET film with different PET substrate thickness when various bending radius of curvature are applied.

The experiment details of the patterned ITO/PET films proposed in this study are listed in Table 2. Different bending radii of curvature (i.e., 2, 4, 6, 8, and 10 mm) were selected to investigate the limitation of flexible content under a convex-type bending load. The ITO transparent conducting film was subjected to tensile stress under a convex-type bending load. To ensure the measurement accuracy of the proposed experiment, three specimens of the patterned ITO/PET film was tested for each experimental condition, and each film was measured five times to obtain the average electrical resistance and the standard deviation. Figure 6 shows the normalized resistance of the tested ITO/PET sample for PET substrate thickness of 50, 100, and 125 µm. The results show that the resistance was significantly increased when a bending radius below 4 mm was applied, regardless of the thickness of the PET substrate. In addition, the measured resistance of the pattern 2 ITO/PET film with narrow line width displayed a larger resistance change than the pattern 1 ITO/PET film, implying that the decrease in ITO line width leads to the failure of the patterned ITO/PET film because of the reduced moment of inertia. Thus, the pattern influence of the ITO/PET film was clarified by the experimental data. This study also proposed a simulation approach using FEA. The stress–strain distribution of the patterned ITO/PET film was simulated to validate the trend of the measured electrical resistance data. The failure criteria of the ITO maximum tensile bending strain were used to estimate the mechanical reliability of the patterned ITO/PET film.

## 4. Stress–Strain Simulation Approach of Patterned ITO/PET Thin Films

**Figure 7:**
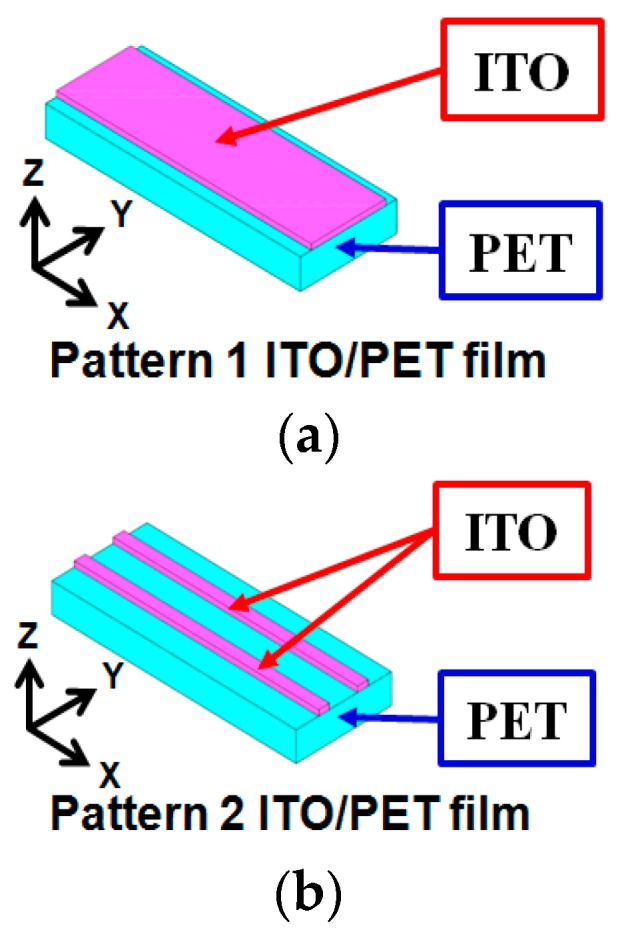
Finite element analysis (FEA) model of patterned ITO/PET film utilized in stress–strain simulation: (**a**) pattern 1 (ITO width = 3.92 mm); (**b**) pattern 2 (ITO width = 0.37 mm).

The fracture of brittle transparent conducting films on flexible substrates is a critical reliability issue in the structure of stacked thin films. The stress–strain distribution of the patterned ITO/PET film was simulated to estimate the failure possibility and critical locations of the ITO/PET film under various bending loads. The FEA models of the patterned ITO/PET films are presented in Figure 7. The patterned ITO/PET film had the following characteristics: 150 nm-thick ITO and three different PET substrate thicknesses (50, 100, and 125 µm). Table 3 lists the material properties utilized in the stress–strain simulation.

### 4.1. Stress Distribution of Patterned ITO Film Deposited on a Compliant PET Substrate

**Figure 8:**
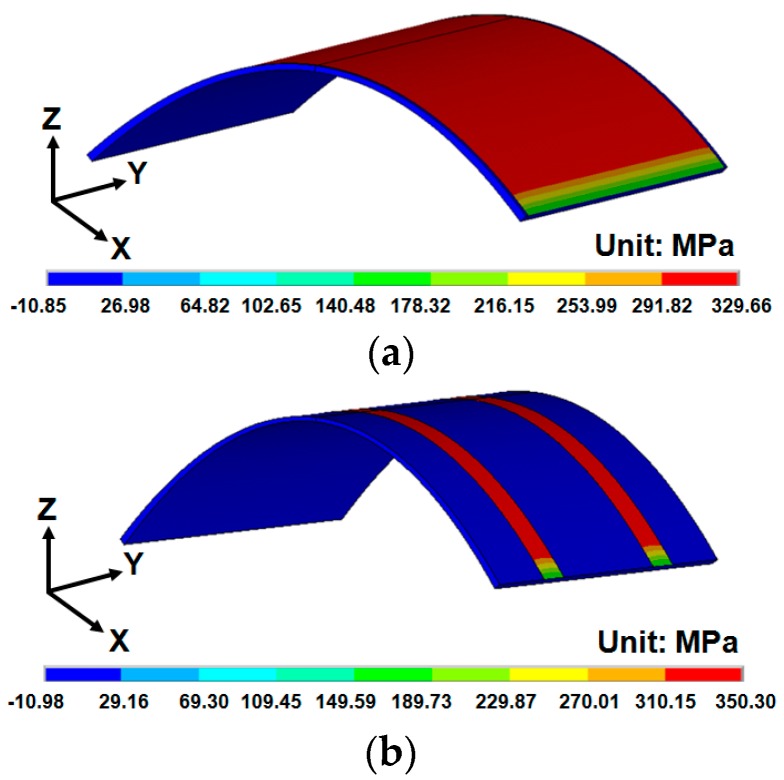
Bending stress contour of patterned ITO/PET film with 50 µm substrate thickness under a bending radius of curvature = 10 mm: (**a**) pattern 1 (ITO width = 3.92 mm); (**b**) pattern 2 (ITO width = 0.37 mm).

To explore the stress distribution of the patterned ITO/PET film, a bending load with 10 mm radius of curvature was applied on the bottom plane of the 50 µm-thick patterned ITO/PET FEA model. As shown in Figure 8a, the maximum tensile bending stress occurred on the top surface of the ITO under convex-type bending load. A stress magnitude of 329.659 MPa was obtained. A similar approach was applied to the pattern 2 ITO/PET film, as shown in Figure 8b. The simulated tensile bending stress was 350.301 MPa. A higher bending stress was observed on the pattern 2 ITO/PET film with narrow line width of 0.37 mm compared to the 3.92 mm of the pattern 1 sample. This phenomenon can be explained as follows. For a 0.37 mm narrow line width of the ITO film, a reduced moment of inertia resulted in a decreased line width of the ITO film. The standard formula of the bending stress calculation can be expressed as follows:

(2) $$\sigma =\frac{My}{I}$$

where σ is the uniaxial bending stress, *M* refers to the bending moment, and *y* and *I* denote the distance to the neutral axis and area moment of inertia, respectively. From Equation (2), the bending stress difference of the pattern 1/pattern 2 ITO/PET films was confirmed by the analytical results. Thus, decent and reliable simulation results were obtained for the estimation of the stress–strain-induced failure of the ITO/PET films.

### 4.2. Thickness Effect of ITO Coating

**Figure 9:**
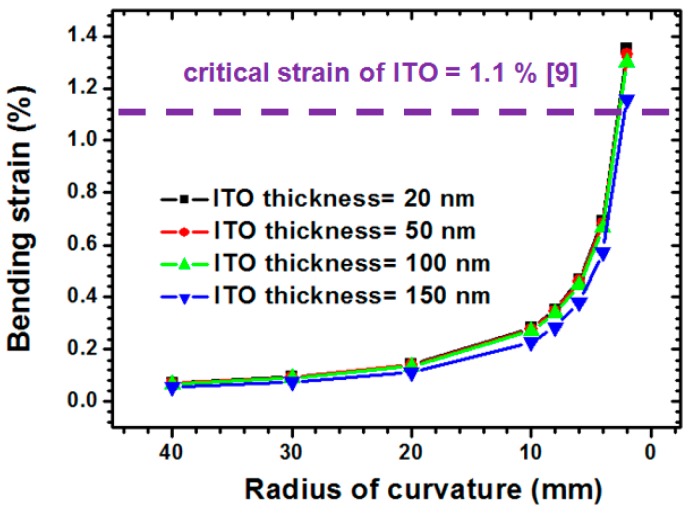
Thickness effect of ITO film coated on PET substrate on induced ITO bending strain estimation.

The influence of various ITO thicknesses was investigated to evaluate the thickness effect of the induced ITO bending strain. Figure 9 shows the simulation results of the pattern 1 ITO/PET film for ITO thicknesses of 20, 50, 100, and 150 nm. The 1.1% critical tensile strain of ITO measured by Chen et al. [9] was selected to estimate the mechanical reliability of the ITO-coating film. A significant induced ITO bending strain was observed when the bending radii ranged from 10 to 2 mm. For a 20 nm-thick ITO film coated on PET substrate with a bending radius of curvature ranging from 40 to 2 mm, the induced bending strain significantly increased from 0.0715% to 1.3531%.

### 4.3. Pattern and Compliant PET Substrate Thickness Influence of ITO/PET Film

**Figure 10:**
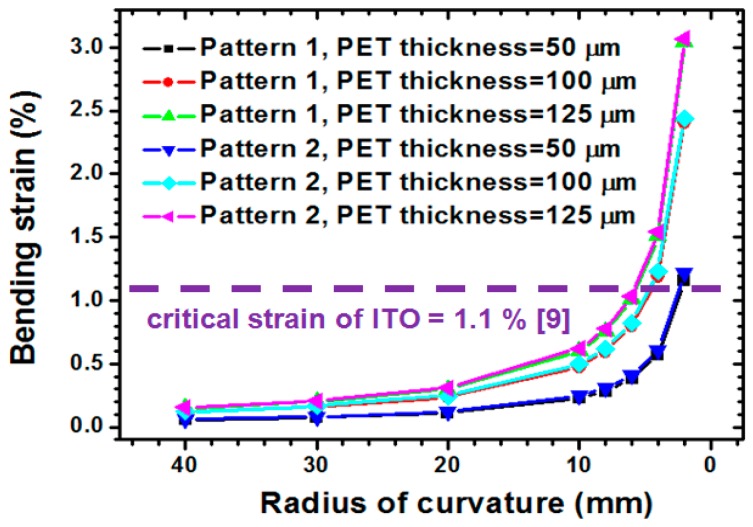
Pattern and PET thickness effects on the variation of ITO bending strain induced by different bending radiuses.

A similar investigation was applied to explore the pattern and PET substrate thickness influence of induced ITO bending strain under various PET substrate thicknesses (50, 100, and 125 µm). As depicted in Figure 10, the thicker compliant PET substrate tends to induce a larger bending strain of the ITO because of the thicker PET substrate, leading to an increased distance from the neutral axis to the ITO surface. Similar to the simulation results shown in Section 4.1, the narrow line width of the ITO pattern resulted in a larger bending strain, because the reduction of the ITO reduced the moment of inertia. However, the induced bending strain of the two types of ITO pattern were slightly different when the same PET thickness and bending radius were considered. When a thinner PET substrate with 50 µm thickness and 2 mm bending radius were considered, the bending strain of pattern 1 and pattern 2 were 3.0466% and 3.0661%, respectively. For the pattern 2 ITO/PET film with a narrow line width of 0.37 mm, the strained induced by bending load increased from 1.218% to 3.0661%, and the PET thickness ranged from 50 to 125 µm. Moreover, a crucial bending radius of 2.5 mm was estimated when the 50 µm-thick PET substrate was achieved. These results clarify the effects of the pattern and the PET thickness on the mechanical reliability of the ITO coating. Therefore, a wider line width and a thinner PET substrate were acquired to enhance the mechanical reliability of the patterned ITO film.

## 5. Conclusions

An electrical resistance experimental method and a stress–strain simulation approach was proposed for ITO/PET films to examine the effects of the pattern and the thickness effect of stacked films under various bending loads. The calculated electrical resistance value agrees well with the experimental data of each patterned ITO/PET film, ensuring measurement accuracy and sample testing without damage. Moreover, the electrical resistance of the patterned ITO/PET film was significantly increased when a bending radius of curvature below 10 mm was considered. According to the experimental data, a narrow line width tends to accelerate the brittle failure of the ITO film. Thus, a wider line width was suggested for the ITO film to avoid failure. Furthermore, this study also evaluates how the thickness of the PET substrate affects the changes in electrical resistance under a bending load. The results indicated that the patterned ITO film deposited on a thicker PET substrate was easily cracked because of the significant increase in the distance from the neutral axis to the surface of the ITO film. Moreover, the experimental data of the critical tensile strain were analyzed by stress–strain simulation to determine the mechanical reliability of the ITO film coated on a PET substrate. The significant ITO bending strain obtained when the bending radius of curvature ranged from 10 to 2 mm dominated the stress–strain magnitude on the surface of the ITO film. Meanwhile, the pattern influence of the ITO film clarified that the narrow line width tends to induce a larger bending strain because of the reduction of the moment of inertia. In addition, ITO and PET thickness also play important roles in enhancing the mechanical reliability. Thus, a thicker ITO and thinner PET thickness was preferred to avoid the brittle failure of the ITO film.

## Figures and Tables

**Table 1:** List of sheet resistance, theoretical value, and measurement results of original and cut patterned ITO/PET sample.

PET Thickness	Sheet Resistance	Pattern 1		Pattern 2	
		Cal. (kΩ)	Mea. (kΩ)	Cal. (kΩ)	Mea. (kΩ)
50 µm	120 ± 20 Ω/ϒ	3.02–4.23	3.57	8.78–12.29	10.15
100 µm	140 ± 30 Ω/ϒ	3.33–5.14	4.26	9.66–14.92	10.67
125 µm	150 ± 30 Ω/ϒ	3.63–5.44	4.95	10.53–15.80	13.03

**Table 2:** Experiment conditions designs of patterned ITO/PET film.

Experimental Conditions	Radius of Curvature (mm)							
	2	4		6		8		10
	Convex Bending							
Pattern Design	Pattern 1 (Line width = 3.92 mm)				Pattern 2 (Line width = 0.37 mm)			
PET Thickness (μm)	50		100				125	

**Table 3:** Material properties used in stress–strain simulation of patterned ITO/PET film.

Materials	Young’s Modulus (GPa)	Poisson’s Ratio
ITO [21]	118	0.3
PET [22]	3.1	0.4
